# Using zebrafish to study the function of nephronophthisis and related ciliopathy genes

**DOI:** 10.12688/f1000research.15511.2

**Published:** 2018-12-03

**Authors:** Elisa Molinari, Simon A. Ramsbottom, Veronica Sammut, Frances E. P. Hughes, John A. Sayer

**Affiliations:** 1 Institute of Genetic Medicine, Newcastle University, Newcastle upon Tyne, NE1 3BZ, UK; 2 Renal Services, Newcastle upon Tyne Hospitals NHS Foundation Trust, Newcastle upon Tyne, NE7 7DN, UK; 3 NIHR Newcastle Biomedical Research Centre, Newcastle upon Tyne, NE4 5PL, UK

**Keywords:** Kupffer’s vesicle; acetylated alpha-tubulin, primary cilia, somite

## Abstract

Zebrafish are a valuable vertebrate model in which to study development and characterize genes involved in cystic kidney disease. Zebrafish embryos and larvae are transparent, allowing non-invasive imaging during their rapid development, which takes place over the first 72 hours post fertilisation. Gene-specific knockdown of nephronophthisis-associated genes leads to ciliary phenotypes which can be assessed in various developmental structures. Here we describe in detail the methods used for imaging cilia within Kupffer’s vesicle to assess nephronophthisis and related ciliopathy phenotypes.

## Introduction

The zebrafish, *Danio rerio*, is a powerful model in which to study inherited diseases ^1, 2^. A total of 70% of human genes have a zebrafish orthologue, which can be exploited in this model to determine expression and function ^3^. Zebrafish can be used in a huge variety of manipulations, including forward genetic screens, targeted gene editing and pharmacological screening of new compounds. Here we describe techniques to visualize cilia within the developing node called Kupffer’s vesicle (KV) ^4^ using fluorescence microscopy. These techniques can be successfully utilized to study nephronophthisis and related ciliopathy (NPHP-RC) genes ^5– 7^.

Nephronophthisis (NPHP) is an autosomal recessive inherited cystic kidney disease and a common cause of childhood and adolescent end-stage renal disease ^8^. Genetically the disease is heterogeneous, with more than 20 genes involved in pathogenesis ^9, 10^. Notably, extra-renal manifestations are seen in 15% of patients, including disorders of the brain, retina, heart, liver and skeletal system. Remarkably, all genes known to cause NPHP and its associated syndromes express their protein products in the primary cilium, basal body or centrosome, leading to the umbrella term NPHP-RC for this group of conditions ^11^. The disease pathogenesis of NPHP-RC is intimately related to primary ciliary structure and function (in terms of both sensing and signalling). Studying these diseases and how they affect the cilia (especially during development) has led to important mechanistic insights ^12^.

KV in the zebrafish is a ciliated organ that functions in an analogous manner to the murine embryonic node ^13, 14^ and is important for the establishment of left-right (LR) asymmetry. The KV forms at around 10–12 hours post-fertilization (hpf) from a cluster of dorsal forerunner cells that have migrated to the tail bud. KV cells are ciliated and cilia mediated flow establishes LR body patterning and organ asymmetry. Typically there are around 50–60 cilia within the KV, of length 3–5 µm ^4, 15^. KV cilia are a mixture of motile and immotile. Changes in cilia number, motility and length can have a significant impact on the generation of flow and hence laterality (KV function) ^16– 18^. Despite being a well-defined structure, locating and imaging KV is challenging and we hope to demystify this in the detailed methodological review presented here.

We aim to provide a precise methodological approach for the identification and staining of KV in developing zebrafish embryos. We hope to provide details of how to exploit this ciliated structure as a readout and disease model for inherited ciliopathy syndromes.

## Methods

### Materials

1. Phosphate buffered saline (PBS; 10x): 25.6 g Na _2_HPO _4_·7H _2_O, 80 g NaCl, 2 g KCl, 2 g KH _2_PO _4_. Make up to 1 litre with H _2_O and autoclave at 121°C.
2. 4% Paraformaldehyde (PFA) (Sigma-Aldrich ; catalogue number 16005) in PBS ( note 1).
3. Methanol (VWR; catalogue number 20846.326).
4. Acetone (Sigma-Aldrich; catalogue number 24201).
5. Blocking solution: 5% Bovine serum albumin (BSA) (Sigma-Aldrich; catalogue number A7906) in PBS containing 0.01% Tween-20 (Sigma-Aldrich; catalogue number P9416) and 0.1% DMSO (Sigma-Aldrich ; catalogue number D8779).
6. Glass vials and lids (Qmx laboratories; catalogue number V0040, V0309).
7. Microscope slides (VWR; catalogue number 631-1550).
8. PVC insulation tape (Onecall; catalogue number CBBR7213) ( note 7).
9. Primary antibodies for ciliary and KV staining e.g. anti-acetylated α-tubulin (Sigma-Aldrich; catalogue number T6793); anti-aPKC (1:500, SCBT) Santa Cruz Biotechnology; sc-216).
10. Secondary antibodies e.g. Alexa Fluor® 488 Donkey Anti-Mouse IgG Antibody (Thermo Fisher Scientific; catalogue number A-21202); Alexa Fluor® 568 Donkey Anti-Rabbit IgG Antibody (Thermo Fisher Scientific; catalogue number A10042).
11. Mounting medium (Vectashield H-1200, Vector Laboratories).

### Fixing for KV imaging

Imaging KV allows for easy analysis of cilia structure and function. It can be initially seen from the 5 somite stage (ss), but is most easily visualized between the 8 and 12 ss as it increases in size over this developmental window ( Figure 2 and note 2).

**Figure 1.**
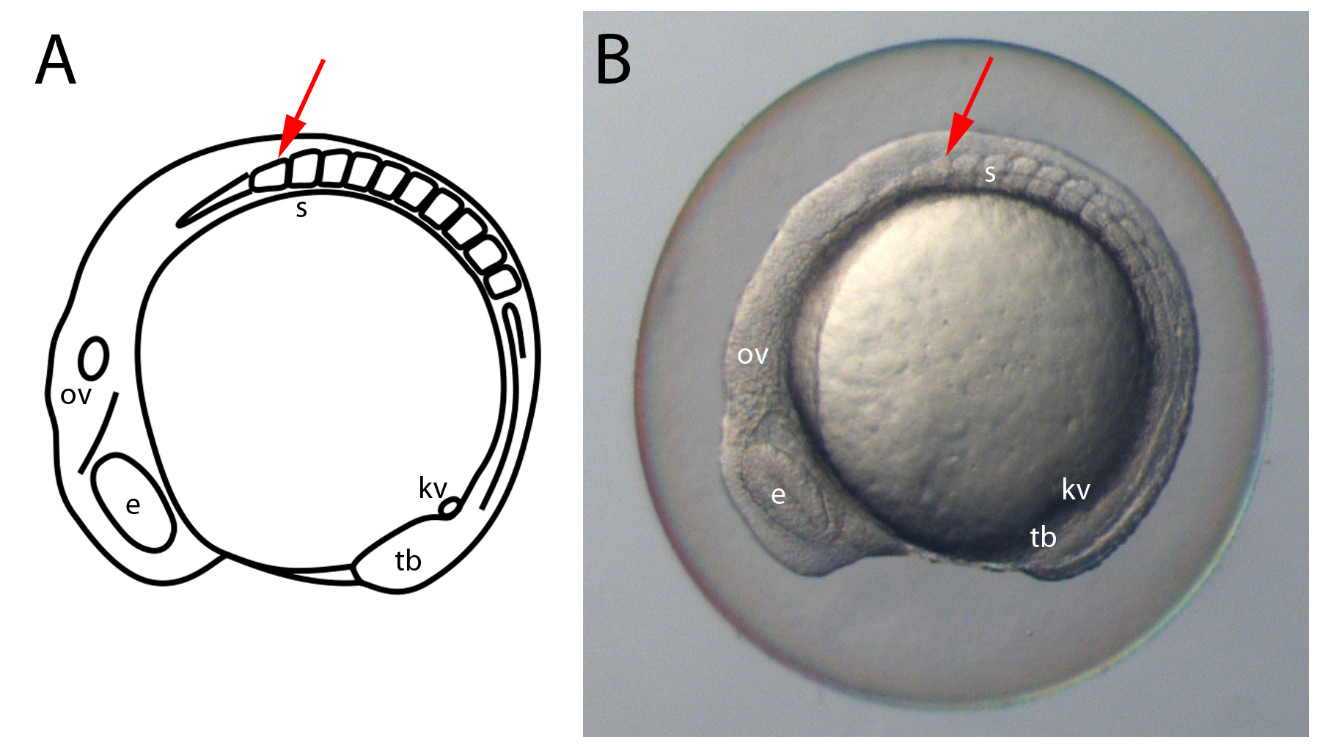
Staging embryos by somite counting. A schematic ( **A**) and light-microscopy image ( **B**) showing a lateral view of a 10 somite stage zebrafish embryo. The most anterior somite (red arrow) is slightly shorter and broader than more posterior somites. The generation of somites is tightly linked to the overall development of the embryo and therefore allows for accurate aging. e, eye; kv, Kupffer’s vesicle; ov, otic vesicle; s, somites; tb, tail bud.

**Figure 2.**
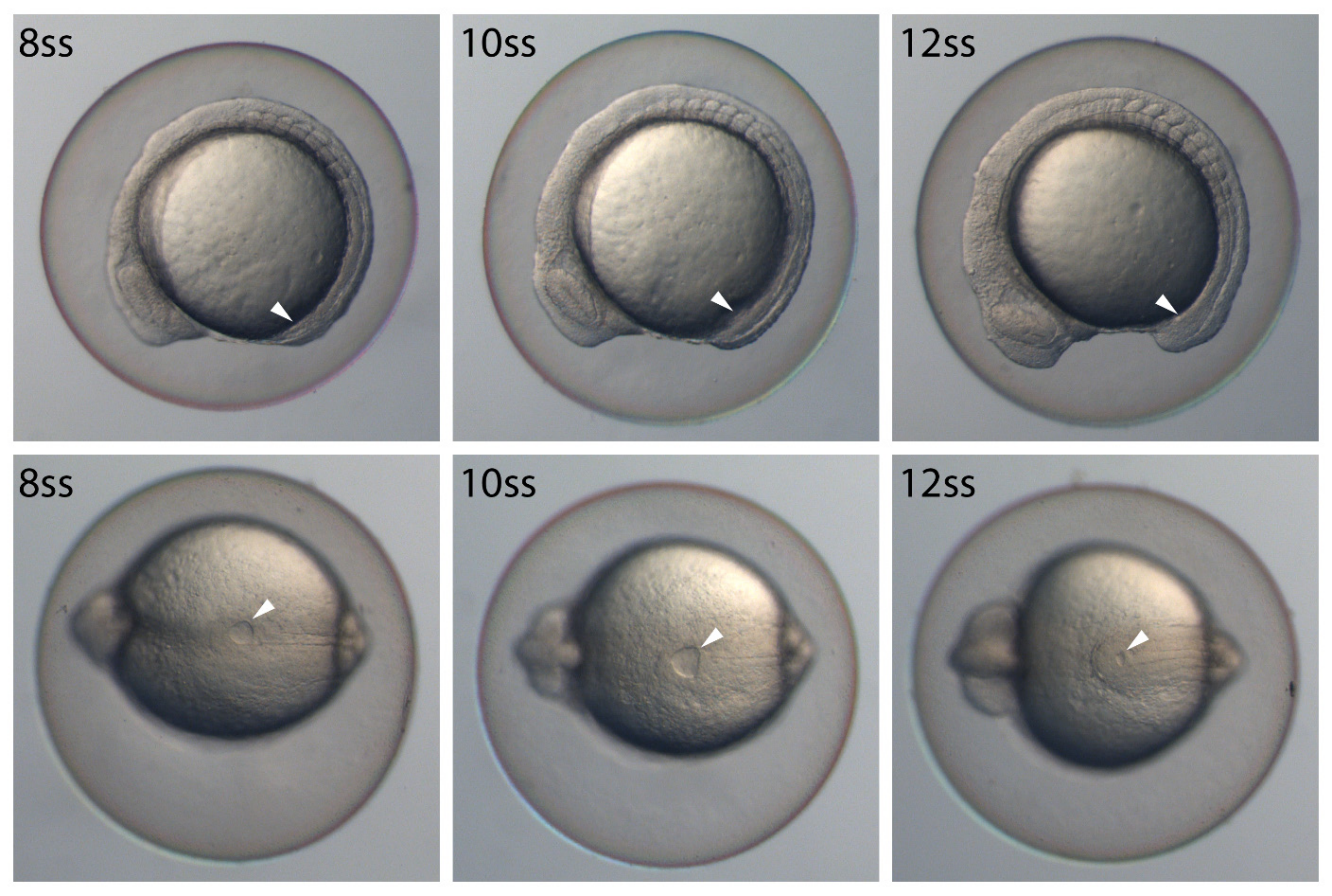
Kupffer’s vesicle changes significantly in a short developmental window. Lateral (top panels) and ventral (bottom panels) views of zebrafish from the 8 somite stage (ss) to the 12 ss. Over this developmental window, Kupffer’s vesicle (white arrowhead) expands and then shrinks, reaching its greatest size at the 10 ss. At 28.5°C, zebrafish will form approximately two somites per hour, so the above panels represent a 4-hour period.

To assess the age of the fish embryos, roll the embryo on to its side and count along the number of fully-formed somites, starting from the head to the tail ( Figure 1). At the 10 ss the tail will begin to bud off from the yolk and this can be used as a secondary measure ( Figure 2).

1. Fix 8-12 ss Zebrafish embryos in 4% paraformaldehyde in phosphate buffered saline (4% PFA in PBS) overnight at 4°C. Do not remove the chorions before fixation ( note 3).
2. Dehydrate the embryos in Methanol in a graded fashion from 25% to 100% methanol (in distilled water) in steps of 25%, changing the wash every 10 minutes.
3. Store the embryos in 100% Methanol at -20°C until required.

### Antibody staining for cilia in KV

Throughout this procedure you should use glass vials and pipettes ( note 4).

1. Rehydrate embryos slowly through a graded series of Methanol from 100% to 0% (in distilled water), changing the wash every 5 minutes. Keep embryos on ice throughout ( note 5).
2. Wash twice more with distilled water to remove all traces of methanol
3. Incubate in chilled acetone at -20°C for 5 minutes, to permeabilise the tissue
4. Replace acetone with distilled water
5. Wash twice more with distilled water to remove all traces of acetone
6. Replace water with PBS (to reduce osmotic stress in de-chorionated tissues)
7. Manually de-chorionate the embryos using watchmaker’s forceps or Green 21 gauge needles
8. Transfer embryos into 2ml glass vials
9. Incubate embryos in 1 ml blocking solution for 2 hours
10. Incubate embryos in primary antibody in blocking solution overnight at 4°C ( note 6)
11. Wash embryos 3 times in PBS for 15 minutes or longer per wash
12. Incubate embryos in secondary antibody in blocking solution overnight at 4°C
13. Wash embryos 3 times in PBS for 15 minutes or longer per wash

At the end of the staining procedure, embryos can be stored for up to two weeks at 4°C in PBS, or long-term at 4°C in 2% PFA in PBS. Staining of embryos does not appear to be significantly diminished by longer-term storage.

### Imaging KV

Once zebrafish embryos have been stained, the easiest way to visualize KV is by mounting the tail only in relief slides. Many tails can be imaged on a single slide, making slide preparation, imaging and storage easier.

**Figure 3.**
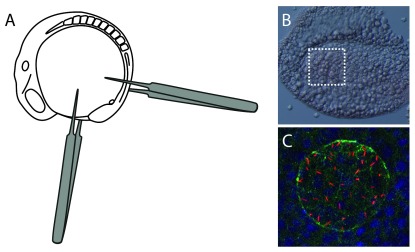
Mounting samples for visualization of Kupffer’s vesicle. Schematic showing a 10 somite stage zebrafish embryo ( **A**). Forceps can be used as depicted to separate the tail tip from the rest of the embryo. Excess yolk is then removed, and the tail tip mounted flat on to a microscope slide. ( **B**) Bright field image overlaid with fluorescence image of flat-mounted tail tip. The white box depicts the location of Kupffer’s vesicle. Fluorescence from acetylated-α tubulin (red) can be seen within this region. ( **C**) Confocal image of Kupffer’s vesicle, visualised using acetylated-α tubulin (red), aPKC (green) and DAPI (blue).

Prepare slides
  1. Take a glass slide and lay one layer of PVC tape on to it ( note 7).
  2. Cut a rectangular hole in the middle of the tape and remove this portion to create a well.
Prepare and mount embryos
  1. Remove the tail tip of the embryos using forceps ( note 8 and Figure 3)
  2. Place up to 10 tail tips into the prepared well
  3. Orient the tail tips so that they face upwards
  4. Carefully remove the surrounding PBS ( note 9)
  5. Fill the well with mounting medium ( note 10)
  6. Place a cover slip over the well and seal the edges with nail varnish

Imaging can be undertaken with any fluorescent microscope that is able to remove out-of-focus light. While the KV can be seen with a traditional global excitation and capture system, there is too much noise resulting from the surrounding tissue. A confocal microscope offers the best image quality, but optical sectioning using structured illumination also gives good results. The staining and mounting method described are suitable for both upright and inverted microscopy.

Following capture, images can be analysed for relevant parameters using FIJI (ImageJ) software. To measure the length of cilia use the segmented line tool on a maximum intensity projection of a z-stack. KV volume can be calculated using the area delineated by aPCK; to obtain a good approximation of the average radius, use the oval tool to outline the KV and measure the area. Note that although the KV may appear as an ellipse in the section, it should be modelled in 3D as a sphere. Using the area of the KV in 2D, calculate the average radius ( $r=\sqrt{A}/\pi$) and subsequently the volume ( $V=\frac{4}{3}\pi r^{3}$).

### Validation

Light microscopy images were acquired using a Leica MZ16F stereo microscope and Leica Application Suite V4.2. The presence of fluorescently labelled acetylated-α tubulin was confirmed in flat-mounted embryos using a Leica MZ16F stereo microscope with a Leica external light source for fluorescence excitation EL6000 with a red fluorescence protein filter.

Fluorescent microscopy images were captured using an Axio Imager Z1 fluorescent microscope (Zeiss), using an apotome for optical sectioning and a Colibri light source and filter sets for DAPI (blue), Alexa Fluor488 (green) and Alexa Fluor 594 (Red) with a 20X air objective.

Zebrafish were maintained in a Home Office aquarium facility at a temperature of 28°C. All zebrafish procedures were performed under Home Office UK license regulations.

### Notes

**1. Preparation of paraformaldehyde**

Paraformaldehyde is dangerous in its powdered form and should be handled with care using a fume hood and mask. To successfully get PFA into solution it is necessary to warm it to 60°C and add sodium hydroxide to raise the pH. At the correct temperature and pH (pH 7), the powder will go into solution rapidly. After cooling check the pH is correct. You can freeze aliquots (e.g. 10–20 ml) of PFA for later use. Once thawed, keep at 4°C for up to three days.

**2. Fixing zebrafish at the 10 ss**

At 28.5°C, embryos reach the 10 ss approximately 14 hpf, making collection difficult (as this is typically very late at night, assuming fish lay eggs in the morning in a standard aquarium with unmodified night/day cycles). To facilitate collection of the 10 ss embryos, it is possible to slow the development of the embryos down by placing them at a slightly lower temperature. At around 24°C the embryos will not reach the 10 ss until approximately 21 hpf (i.e. the following morning).

**3. Embryo fixation**

It is usual to remove the chorion before fixation of fish embryos. At early stages, however, the embryos are very fragile and also the yolk tends to stick to almost anything when rehydrated. Thus it is best to leave the chorion on when undertaking the rehydration procedure, prior to immunostaining.

**4. Immunostaining in glass**

Embryos should be transferred using glass pipettes; they will stick to plastic and disintegrate easily. Immunostaining should be carried out in glass vials only. Using 2-ml glass vials allows for staining in a small volume of liquid (≤200 µl) which helps to reduce reagent costs and reduces the storage space required. Typical volumes are 2 ml for washes and 200 μl of antibody solution.

**5. Embryo rehydration**

Embryos are very fragile at this stage and should be handled with care. When rehydrating, only remove 50% of the solution they are in and replace with the next one (e.g. remove half of the 100% methanol, and replace with half of 75% methanol to give 87.5%, then remove most of this and place into 75% methanol such that the steps are of roughly 12.5%). Mixing methanol with water leads to an exothermic reaction so embryos should be kept on ice throughout to maintain their low temperature.

**6. Primary antibodies**

Any antibody which specifically detects ciliary proteins can be used for analysis of KV, provided that it cross-reacts with zebrafish antigen. Staining of the intra-flagellar transport (IFT) machinery, the axoneme, or the ciliary membrane are all possible with this technique and allow for analysis of both structural and functional defects within zebrafish cilia. For simple detection of cilia structure the best antibody is anti-acetylated-α tubulin (Sigma-Aldrich; catalogue number T6793) at 1:500 for the ciliary axoneme. To visualise KV epithelium, counterstain with anti-aPKC at 1:500.

**7. Generation of imaging chamber**

While it is possible to purchase slides with wells of varying sizes, most commercially available slides have a circular bottom making it difficult to get a uniform distance from the cover slip to the sample. As a result only one or two samples can be placed in each. To generate a uniformly flat imaging surface, PVC tape can be used to convert any flat slide to a relief slide in which you can create an imaging chamber to fit your own size requirements. For KV visualization, one layer of tape provides ample depth to prevent the sample being crushed, while keeping the sample close to the cover slip. Detailed information and a visual demonstration of imaging chamber generation has been reported previously ^19^.

**8. Embryo dissection for KV visualization**

To get a good image of KV it is best to remove the tail tip from the rest of the embryo and lay it flat. Using two pairs of forceps, pinch two-thirds of the way along the back of the embryo, to separate the posterior part of the embryo from the anterior, and between the head and tail to detach the tail from the yolk sac. Remove as much yolk from the remaining tissue as possible.

**9. Removal of PBS from the relief slide**

Due to the small size of the dissected zebrafish tail tip, it can be difficult to remove the PBS from the imaging chamber without disturbing the samples. To facilitate this process, use a razor blade to cut off a medium-sized (20–200 µl) plastic micropipette tip at 45°. The cut tip can be placed flat on to the microscope slide allowing for aspiration of PBS without disturbing the samples.

**10. Mounting medium**

Mounting medium with an anti-fading agent is essential for the successful imaging of immunofluorescence-stained KV. Some mounting media is available in a hard-setting format e.g. Vectashield H-1500, but it is preferable to use a non-setting medium such as Vectashield H-1200.

Raw, uncropped images from which Figures 1-3 were generated Leftmost image, Figure 1; middle six images, Figure 2; rightmost two images, Figure 3. Click here for additional data file. Copyright: © 2018 Molinari E et al. 2018 Data associated with the article are available under the terms of the Creative Commons Zero "No rights reserved" data waiver (CC0 1.0 Public domain dedication).

## Discussion

The use of zebrafish for modelling genetic diseases has proven to be very significant. Zebrafish are advantageous given the presence of transparent developmental stages. They are straightforward to maintain and genetic manipulations can be performed with relative ease. Here we have focused on accessing and imaging the zebrafish KV, a transient developmental structure with a lumen and ciliated epithelial cells lining it. The KV functions, via its cilia, serves as a ‘left-right’ organiser in order to determine left-right asymmetry within the zebrafish embryo ^13, 14^. Within the KV, a single layer of epithelial cells surround a fluid-filled lumen into which they extend a motile cilium, generating asymmetric fluid flows that allow left-right patterning signals to be transmitted. Thus the KV is a simple yet accessible structure that is ideal for investigating cilia structure and function, and their role in inherited human ciliopathy syndromes. Nearly all new ciliopathy syndromes described in man use zebrafish, and their KV in particular, to gain insights into disease pathogenesis (use of zebrafish as a model for human ciliopathies is reviewed in Song *et al*., 2016 ^2^). Genetic knockdowns can cause defects in KV that may affect the size and structure of the organ as well as the cilia, and RNA rescue can determine the specificity of this effect. The study of KV therefore provides an accessible tool for the modelling of ciliopathies and interventions, such as genetic therapies and pharmacological agents ^20^, which may be used to rescue underlying cilia defects.

## Data availability

The data referenced by this article are under copyright with the following copyright statement: Copyright: © 2018 Molinari E et al.

Data associated with the article are available under the terms of the Creative Commons Zero "No rights reserved" data waiver (CC0 1.0 Public domain dedication). 

**Dataset 1. Raw, uncropped images from which Figures 1- 3 were generated.** Leftmost image, Figure 1; middle six images, Figure 2; rightmost two images, Figure 3. DOI: 10.5256/f1000research.15511.d210727 ^21^.
