# Supplementary material for: Using zebrafish to study the function of nephronophthisis and related ciliopathy genes
Source: F1000Res. 2018 Dec 3;7:1133. Originally published 2018 Jul 25. [Version 2] doi: 10.12688/f1000research.15511.2 (PMC6127739; doi:10.12688/f1000research.15511.2)
Supplement: Peer review history

# Peer review: Referee response for version 2

**DOI:** 10.5256/f1000research.18693.r41419

**Published:** 2019-01-28

**Authors:** Weibin Zhou

**Affiliations:** 1 Duke University, Durham, NC, USA

The revision has addressed all of my concerns.

I have read this submission. I believe that I have an appropriate level of expertise to confirm that it is of an acceptable scientific standard.

# Peer review: Referee response for version 2

**DOI:** 10.5256/f1000research.18693.r41420

**Published:** 2018-12-18

**Authors:** Glenn P. Lobo

**Affiliations:** 1 School of Medicine, Department of Ophthalmology, Medical University of South Carolina (MUSC), Charleston, SC, USA

This reviewer is satisfied with the additional revisions made.

I have read this submission. I believe that I have an appropriate level of expertise to confirm that it is of an acceptable scientific standard.

# Peer review: Referee response for version 1

**DOI:** 10.5256/f1000research.16913.r37382

**Published:** 2018-09-05

**Authors:** Roslyn Simms

**Affiliations:** 1 University of Sheffield, Sheffield, UK

Molinari et al. present a well written and clear description of the methodology required to image the transient developmental zebrafish organelle, Kupffer's Vesicle (KV). Overall, they clearly show KV development using light microscopy and a confocal image of cilia in KV stained using acetylated-alpha tubulin. 

 Their method will definitely facilitate research to study the structure of KV and its cilia. They provide useful tips on how to overcome practical challenges. However it would be informative to include information on how to measure cilia length (for example software used) and how to calculate KV volume.

 Similarly, additional methodological information will have to be added relating to studying the "function of KV", perhaps using live confocal imaging to study cilia motility, or this comment amended.

 Minor points, which would help the reader:

- I suggest delete "easy" in the Methods section on "Fixing for KV imaging" as this relatively contradicts the challenge of imaging KV which you describe in the introduction.
- Consider including a magnified image of the white arrowed area of the lateral views in Figure 2.
- Add information on how you identified the location of KV from the bright field image in Figure 3B. 
- In the notes section, add the number of KV you advise mounting on each slide for imaging.
- Finally in the discussion, add some references to support your statements:"Nearly all new cilioopathy syndromes,,," and "genetic therapies and pharmacological agents" (the time window to do this will be relatively short). 

Overall an informative methodological article for the zebrafish research community.

I have read this submission. I believe that I have an appropriate level of expertise to confirm that it is of an acceptable scientific standard.

# Peer review: Referee response for version 1

**DOI:** 10.5256/f1000research.16913.r37381

**Published:** 2018-08-30

**Authors:** Glenn P. Lobo

**Affiliations:** 1 School of Medicine, Department of Ophthalmology, Medical University of South Carolina (MUSC), Charleston, SC, USA

Molinari and colleagues present a detailed methodology to functionally access cilia structure in KV vessels of zebrafish.

 I am especially impressed with the details provided, methodology setup and the clarity of the images in this manuscript.

 Suggestions:

1. It would be beneficial to readers if authors provide an image of the "relief slides/ imaging chamber" used for mounting.
2. What type of microscope was used? Upright or inverted? An image of the actual zebrafish in the imaging chamber/ slides on the mounting stage of the microscope would also provide additional information about the set-up. What type of objective was used? Air or oil objective? Additional information on microscopy could be provided.
3. A more in-depth detail on optical sectioning using structured illumination (I presume using an Zeiss Apotome 2) would be beneficial.
4. Which program was used to calculate cilia length? Fiji or Image J? Please provide some additional details.

I have read this submission. I believe that I have an appropriate level of expertise to confirm that it is of an acceptable scientific standard, however I have significant reservations, as outlined above.

# Peer review: Referee response for version 1

**DOI:** 10.5256/f1000research.16913.r36493

**Published:** 2018-08-13

**Authors:** Weibin Zhou

**Affiliations:** 1 Duke University, Durham, NC, USA

Molinari et al. presented a detailed description of the method they have developed to assess the function of ciliopathy-related genes using the zebrafish KV as a model system. In particular, they developed this method to image fluorescently labeled KV cilia such that (I assume) the morphology of those cilia can be analyzed. Overall the description is well written but a little more detail is needed to allow the readers to reproduce and use this method in their own studies. My major questions are as follows:

  

1. The KV cilia are either motile and immotile. I think the method in this manuscript is primarily addressing the morphology of cilia, but the authors did not present any method of analyzing the ciliary morphology after the illustrated imaging process. It would be critical for them to present their quantitative analysis of ciliary morphology based on the fluorescent images, to ensure the completeness of this manuscript.
2. It is not mentioned how many embryos were placed into each glass vial for immunostaining and the volume of staining solution for each sample. This is also pertinent to the quantitative analyses, as I wonder about the typical sample size needed for the quantitative analyses.
3. I think the intro and the discussion needs to be edited by a native English speaker to ensure there is no grammatical error.

I have read this submission. I believe that I have an appropriate level of expertise to confirm that it is of an acceptable scientific standard, however I have significant reservations, as outlined above.
